# The genome- and transcriptome-wide analysis of innate immunity in the brown planthopper, *Nilaparvata lugens*

**DOI:** 10.1186/1471-2164-14-160

**Published:** 2013-03-09

**Authors:** Yan-Yuan Bao, Lv-Yu Qu, Dong Zhao, Li-Bo Chen, Hong-Yuan Jin, Liang-Min Xu, Jia-An Cheng, Chuan-Xi Zhang

**Affiliations:** 1 State Key Laboratory of Rice Biology and Ministry of Agriculture Key Laboratory of Agricultural Entomology, Institute of Insect Sciences, Zhejiang University, Hangzhou, 310058, China

**Keywords:** Nilaparvata lugens, Hemimetabolous insect, Genome, Transcriptome, Innate immunity, Gene expression

## Abstract

**Background:**

The brown planthopper (*Nilaparvata lugens*) is one of the most serious rice plant pests in Asia. *N. lugens* causes extensive rice damage by sucking rice phloem sap, which results in stunted plant growth and the transmission of plant viruses. Despite the importance of this insect pest, little is known about the immunological mechanisms occurring in this hemimetabolous insect species.

**Results:**

In this study, we performed a genome- and transcriptome-wide analysis aiming at the immune-related genes. The transcriptome datasets include the *N. lugens* intestine, the developmental stage, wing formation, and sex-specific expression information that provided useful gene expression sequence data for the genome-wide analysis. As a result, we identified a large number of genes encoding *N. lugens* pattern recognition proteins, modulation proteins in the prophenoloxidase (proPO) activating cascade, immune effectors, and the signal transduction molecules involved in the immune pathways, including the Toll, Immune deficiency (Imd) and Janus kinase signal transducers and activators of transcription (JAK-STAT) pathways. The genome scale analysis revealed detailed information of the gene structure, distribution and transcription orientations in scaffolds. A comparison of the genome-available hemimetabolous and metabolous insect species indicate the differences in the immune-related gene constitution. We investigated the gene expression profiles with regards to how they responded to bacterial infections and tissue, as well as development and sex expression specificity.

**Conclusions:**

The genome- and transcriptome-wide analysis of immune-related genes including pattern recognition and modulation molecules, immune effectors, and the signal transduction molecules involved in the immune pathways is an important step in determining the overall architecture and functional network of the immune components in *N. lugens*. Our findings provide the comprehensive gene sequence resource and expression profiles of the immune-related genes of *N. lugens*, which could facilitate the understanding of the innate immune mechanisms in the hemimetabolous insect species. These data give insight into clarifying the potential functional roles of the immune-related genes involved in the biological processes of development, reproduction, and virus transmission in *N. lugens*.

## Background

Insects have a powerful innate immune system with which to defend against pathogenic intruders. Innate immune responses have been well documented in the metabolous insect species, especially in dipteran and lepidopteran insects, as they are important to human health and agricultural production. By contrast, little is known about the immune responses in hemimetabolous insects, despite the fact that their destruction of agricultural crops has become increasingly serious in recent years. Understanding the immune mechanisms of hemimetabolous insects, especially the insect pests, is becoming an urgent requirement.

All phloem-feeding hemipteran insects depend on symbiotic microorganisms to support the necessary nutrition, development, reproduction and defense against natural enemies of their host insects [1,2]. The brown planthopper, *Nilaparvata lugens* Stål (Hemiptera: Delphacidae), is the most destructive pest for rice throughout Asia. This insect causes extensive rice damage by sucking rice phloem sap and transmitting plant viruses. As a hemimetabolous insect, *N. lugens* is rich in various symbiotic microorganisms, including an intracellular yeast-like symbiont (YLS) and four bacterial microbe phyla, *Proteobacteria*, *Firmicutes*, *Actinobacteria* and *Bacteroidete*[2]. As the virus vector, *N. lugens* transmits two plant viruses, the rice ragged stunt virus and rice grassy stunt virus, which result in rice ‘grassy stunt’ and ‘ragged stunt’ diseases respectively [3]. In addition, three viruses have been characterized in *N. lugens*, including reovirus, Himetobi P virus and commensal X virus [4], and are most likely asymptomatic to host insects. Recently, we have identified a novel nudivirus from *N. lugens* (unpublished). Nudiviruses are a highly diverse group of large, double-stranded circular DNA viruses which are pathogenic for invertebrates [5]. An interesting question arises: how does this insect host maintain a good balance between the symbiotic microorganisms and foreign pathogens? *N. lugens* is expected to have a precise immune strategy for determining defense strategies against foreign microorganisms or tolerating microbial symbionts.

In our previous study, we obtained a large amount of *N. lugens* transcriptomic datasets using the next-generation high-throughput Illumina sequencing, which provided comprehensive gene expression profiles regarding *N. lugens* development (egg, second and fifth instar nymphs), wing dimorphism (macropterous and brachypterous adults) and sex differences (female and male adults) [6], as well as the intestine-specific expression information in *N. lugens* nymphs and adults [7]. More importantly, we first accomplished *N. lugens* whole genomic sequencing and obtained the gene annotation. A thorough search of the *N. lugens* genome sequence, coupled with the transcriptome datasets, generated the detailed immune-related gene information, which included pattern recognition, signal transduction, modulation, and immune responsive effectors. In this report, we first present an overview of the immune-related genes and their expression specificity in hemimetabolous insects. These data may well be helpful in understanding the innate immune mechanisms of *N. lugens* and in establishing their association with insect development, microbial symbionts, and virus transmission.

## Results and discussion

### Pattern recognition molecules

Peptidoglycan recognition protein (PGRP) and β-glucan recognition protein (βGRP)/gram-negative binding protein (GNBP) are two major protein families that sense foreign microbial infection. PGRP was first isolated from hemolymph of the silkworm, as a pattern recognition receptor which binds peptidoglycan (PGN) and triggers prophenoloxidase activating cascade [8]. PGN presents in the cell walls of almost all bacteria, and is a strong elicitor to activate the innate immune response in insects [9,10]. The PGRP family is conserved from insects to mammals. These molecules share an approximately 160 amino acid domain (PGRP domain), with similarities to bacteriophage T7 lysozyme, a zinc-dependent *N*-acetylmuramoyl-L-alanine amidase [11-14]. The most highly diversified PGRP homologues have been identified in *Drosophila melanogaster*[13]. They are expressed as secreted, cytosolic, or transmembrane forms. According to the enzymatic activity, some non-catalytic PGRPs have been implicated in functions as diverse as signal-transducing receptors, positive regulators and effectors [15], while other PGRPs have amidase activity, cleaving lactylamide bonds between the lactyl group of *N*-acetylmuramic acid and the α-amino group of the L-alanine residues in the step peptide of PGN to eliminate its immunogenicity, thus down-regulating or turning off the immune response in insects [12,16,17]. The amidase type PGRPs conserve the five amino acid residues which coordinate with zinc ions and form a catalytic site in the T7 lysozyme [17,18]. However, the receptor-type PGRPs lack some of these residues.

**Figure 1:**
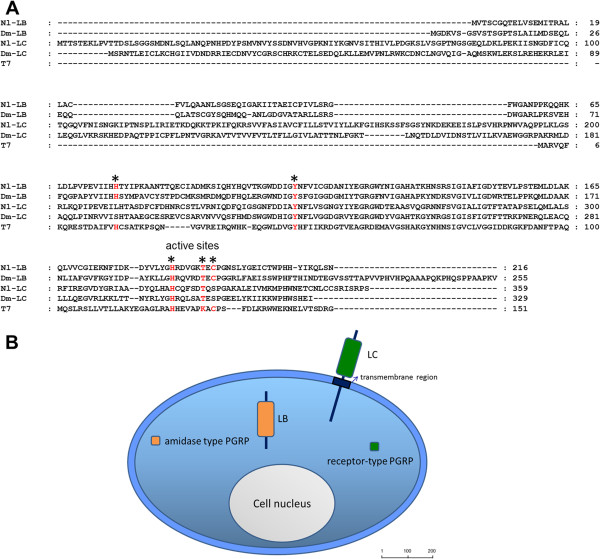
**(A) Multiple alignments of PGRPs and T7 lysozyme.** The ClustalX program was used for alignments. The GenBank accession numbers for the sequences are as follows: *N. lugens PGRP-LB* (**KC355211**); *N. lugens PGRP-LC* (**KC355212**); *D. melanogaster PGRP-LB* (**AFH06370**); *D. melanogaster PGRP-LC* (**ACZ94668**) and *Enterobacteria phage T7 lysozyme* (**AAB32819**). Five amino acid residues required for amidase activity are marked by asterisks and shown in red. **(B)** Predicted cellular distribution of *N. lugens* PGRPs. *N. lugens* PGRP-LC is likely a receptor protein due to its transmembrane region. PGRP-LB lacks the signal peptide and transmembrane region, thus possibly making it a cytosolic protein. The potentially catalytic or non-catalytic amidase activity of the PGRP proteins is shown in orange and green respectively. The size bar indicates the amino acid residues of the deduced proteins.

In this study, we identified two *PGRP* genes by searching the *N. lugens* genome and transcriptome database with the BLASTX algorithm within a cut-off E-value of 10^-5^. The *N. lugens PGRPs* are two long forms that best matched *D. melanogaster PGRP-LB* and *LC* (Figure 1). A quintet of active site residues is essential for amidase activity in T7 lysozyme: His-17, Tyr-46, His-122, Lys-128 and Cys-130 (Zn-ligands) were conserved in the deduced amino acid sequence of the *N. lugens PGRP-LB* (Figure 1A). However, the indispensable active site residues matching His-17 and Cys-130 in the T7 lysozyme are lacking in the *N. lugens PGRP-LC*. In *D. melanogaster*, several catalytic PGRPs have been demonstrated (SC1A, SC1B, LB, SB1) or predicted (PGRP-SB2, SC2) amidase activity [12,16,19-21], while PRGP-LC and LE were shown to act as receptors for PGN in the Imd pathway [22]. A prediction of molecular structure implied that *N. lugens* PGRPs are likely to have different functions (Figure 1B). PGRP-LB had neither the signal peptide nor transmembrane region, and thus it probably remains in the cytoplasm. Five active site residues conserved in PGRP-LB imply the potential amidase activity and might serve as an intracellular PGN scavenger. *N. lugens* PGRP-LC may have no amidase activity, due to the incomplete active sites in the predicted amino acid sequence. A transmembrane region was presented in PGRP-LC, suggesting that it may act as a transmembrane-PGN receptor.

**Figure 2:**
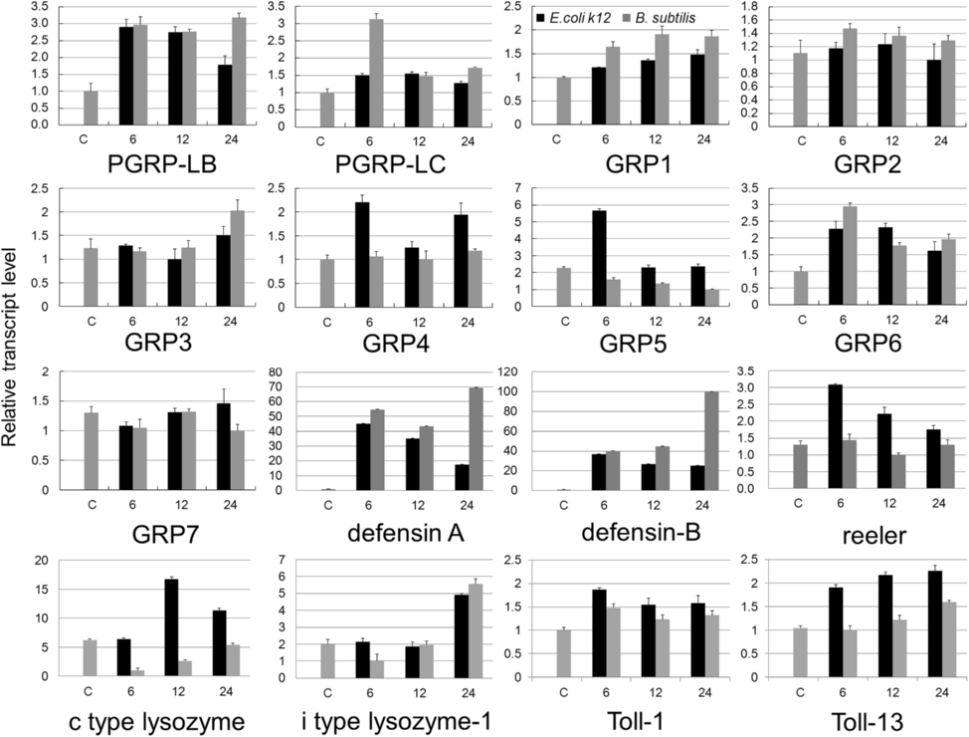
**Responsive expressions to bacterial infection of immune-related genes in *N. lugens *nymphs.** Fifth instar nymphs were microinjected with *E. coli* K12 or *B. subtilis*. Total RNA was extracted from the nymphs at the indicated times after injection. PBS-injected samples were used as controls. First-strand cDNA (20 ng) was analyzed in each real-time quantitative PCR reaction. The reactions were performed with specific primers for amplifying *PGRP/GRP* genes, immune effector genes and *Toll* genes. The relative expression levels of each gene at different time points were normalized using the *N. lugens* 18 s rRNA threshold cycle (Ct) values, which were obtained for reactions run on the same plate. In each assay, the expression level was normalized to the lowest expression level, which was arbitrarily set to one. Three technical replications (n=3) were conducted and the relative transcript levels at each time point were calculated using the ΔΔCt method. The *E. coli* K12- and *B. subtilis* injected samples are shown on the left (black) and right (dark gray), respectively. C refers to the PBS-injected control. 6, 12, and 24 h refer to RNA extracted from bacteria-injected nymphs at 6, 12, and 24 h p.i.

**Figure 3:**
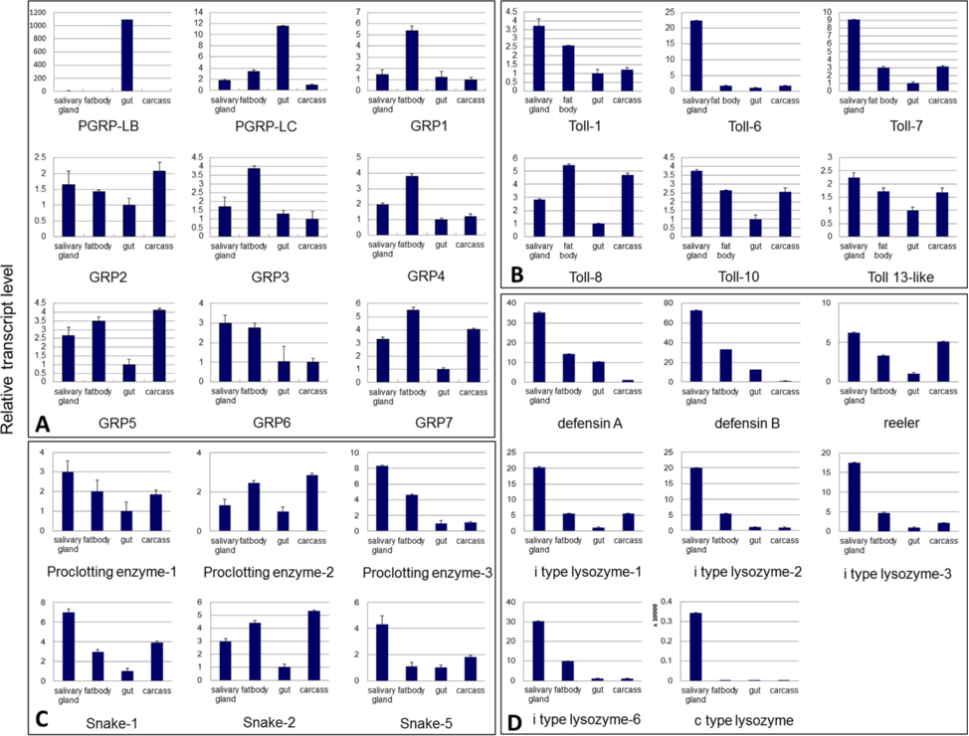
**Tissue specificity of immune-related gene expression in *N. lugens.*** Total RNA was individually extracted from the salivary gland, fat body, gut and the remaining carcass of 5th instar nymphs. First-strand cDNA (20 ng) was analyzed in each qRT-PCR reaction. The reactions were performed with specific primers used to amplify (**A**) *PGRP/GRP* genes; (**B**) *Toll* genes; (**C**) *CLIP* genes; and (**D**) immune effector genes. The relative expression levels of each gene in each tissue were normalized using the *N. lugens* 18 s rRNA threshold cycle (Ct) values which were obtained from reactions run on the same plate. In each assay, the expression level was normalized to the lowest expression level, which was arbitrarily set at one. Three technical replications (n=3) were conducted and the ΔΔCt method was used to measure the relative transcript levels in tissues.

We analyzed the bacteria-induced and tissue-specific expression profiles of *N. lugens PGRP* genes. Immune challenges by heat-killed *E. coli* K12 and *B. subtilis* significantly increased *PGRP-LB* gene expression in *N. lugens* 5th instar nymphs from 6–24 h p.i. *PGRP-LC* gene expression quickly responded to the *B. subtilis* invasion at 6 h p.i; while *E. coli* k12 infection did not significantly increase *PGRP-LC* expression levels during 6–24 h p.i (Figure 2). *PGRP-LB* and *LC* showed very high expression levels in the gut, especially for *PGRP-LB*, which was exclusively expressed in the gut (Figure 3A). These results suggest that *PGRP-LB* and *LC* mainly function in intestinal tracts, a possible route of infection in *N. lugens*. Among insect PGRPs, direct binding to PGN has been demonstrated for *D. melanogaster* PGRP-LB and LC [17]. In *N. lugens*, PGRP-LC may act as a receptor to sense the foreign bacteria that invade the intestinal tract and activate the immune response, while PGRP-LB may be responsible for eliminating the bacteria that enter the cytoplasmic compartment of gut cells. In insect’s innate immune systems, Toll and Imd pathways are turned on following the recognition of PGN by PGRPs, while the removal of immunostimulatory PGN by PGRPs effectively turns off the excess immune responses. We speculated that *N. lugens* PGRP-LB and LC might work in concert with each other to maintain intestinal immune homeostasis.

GNBP and βGRP belong to a pattern recognition receptor family that was initially identified as a component of the proPO-activating cascade in the hemolymph of the silkworm, *Bombyx mori*[23]. GNBP/βGRP had a strong affinity to β-1, 3-glucan of fungi and lipopolysaccharide (LPS) of gram-negative bacteria [24,25], but not to the PGN of gram-positive bacteria. Despite not recognizing for PGN, *D. melanogaster* GNBP1 is required for activating the Toll pathway in response to gram-positive bacterial infections via interaction with PGRP-SA [26,27], while GNBP3 is required to detect fungi and activate the Toll pathway [28]. The GNBP/βGRP family consists of a conserved N-terminal β-1, 3-glucan-recognition domain and a C-terminal β-glucanase-like domain [29,30]. The N-terminal domain plays a crucial role in the detection of pathogens and the activation of insect host defense responses, while the C-terminal glucanase-like domain has neither glucanase activity nor affinity with β-1, 3-glucan, and as such remains an undefined function [31].

**Figure 4:**
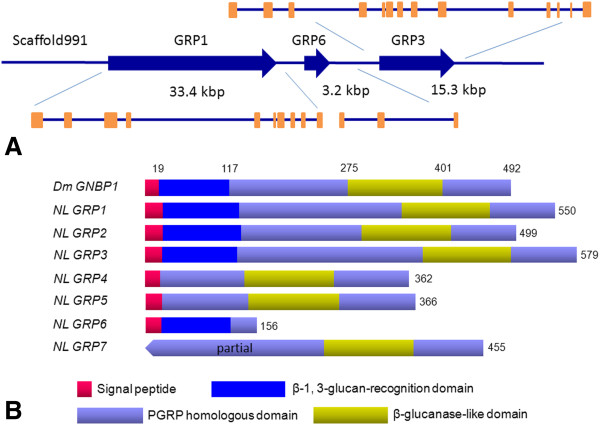
**(A) The gene structure prediction of *N. lugens GRPs*.** The blue arrows indicate the transcription orientations and sizes of *GRP1*, *GRP3* and *GRP6* genes on scaffold991. The exons are shown with orange boxes. (**B**) The schematic representation of *N. lugens GRPs*. The deduced *N. lugens* GRP sequences were compared with *D. melanogaster* GNBP1 (**CAJ18915**). The putative signal peptide, N-terminal β-1, 3-glucan-recognition domain, PGRP homologous domain and C-terminal β-glucanase-like domain are shown in different color boxes. The number indicates the deduced amino acid residues.

**Figure 5:**
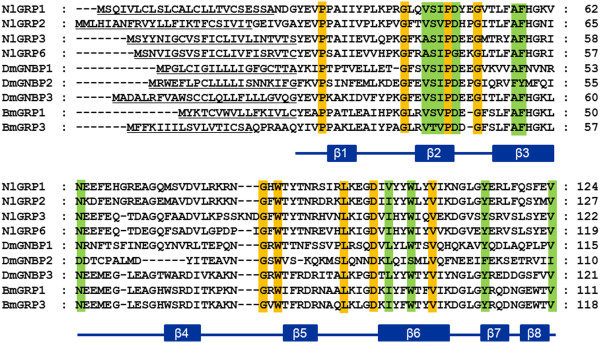
**Alignments of the N-terminal domains of GNBP/GRPs.** The deduced amino acid sequences of *N. lugens* GRPs were compared with *D. melanogaster* GNBP1 (**CAJ18915**), GNBP2 (**CAJ19023**), GNBP3 (**AF228474**), *B. mori* GRP1 (**BAA92243**) and GRP3 (**BAG70413**). The putative signal peptides are underlined. The amino acids in orange and green shade indicate the conserved and type-conserved residues, respectively. The predicted secondary structural elements of eight β-strands are shown below the alignments. DmGNBP, *D. melanogaster* gram-negative bacteria binding proteins; BmGRP, *B. mori* β-1, 3-glucan recognition protein.

In this study, we identified seven *GNBP*/*βGRP* genes in *N. lugens* genome and transcriptome datasets. We designated them as *NlGRP1-7*. These genes consisted of multiple exons. *NlGRP1*, *3* and *6* located at the scaffold991 with the same transcription orientations (Figure 4A & Table 1). A thorough search of the *N. lugens* transcriptome coupled with the RACE method revealed that six genes (*NlGRP1-6*) contained the complete coding regions with the putative signal peptide sequences, implying the secreted proteins (Figure 4B). *NlGRP7* had no signal peptide due to a lack of sequence at the 5^′^ end. A comparison of the deduced amino acid sequences with *D. melanogaster GNBP1* showed that *NlGRP1-3* contained the putative N-terminal β-1, 3-glucan-recognition domain and the C-terminal glucanase-like domain. *NlGRP4* and *5* lacked the N-terminal β-1, 3-glucan-recognition domain, possibly suggesting that they do not directly bind β-1, 3-glucan. By contrast, *NlGRP6* lacked the C-terminal glucanase-like domain. However, the presence of the putative N-terminal β-1, 3-glucan-recognition domain implied its role in the recognition of pathogens. The deduced protein sequences of the *NlGRP1-3* consisted of 499–579 amino acids and showed around 60% of sequence similarities with β-GRP of *Rhodnius prolixus*, while *NlGRP4* and *5* contained approximately 360 amino acid residues, which had 57% sequence similarities with GNBP3 of *Locusta migratoria*. By contrast, *NlGRP6* encodes a small peptide that is composed of 156 amino acids and which showed 64% similarity with β-1, 3-glucan recognition protein of *Bombyx mori*. The N-terminal β-1, 3-glucan-recognition domain was studied rigorously in *D. melanogaster* and *B. mori*. Recently, the secondary structure of the N-terminal domain of *B. mori* GRP was reported, and was found to comprise eight β*-*strands which specifically recognize β-1, 3-glucan [31]. A comparison of the N-terminal domains revealed high sequence similarities among the deduced *N. lugens*, *D. melanogaster* and *B. mori* homologues (Figure 5), suggesting the possible ability of these *N. lugens* GRPs to bind to fungal β-1, 3-glucan.

**Table 1:** **The gene prediction of *N. lugens *pattern recognition molecules**

**Predicted gene**	**GenBank ID**	**Locus**	**Size (aa)**	**Exon**	**Orientation**	**UTR**	**Best match**	**Similarity**	**Mw (KDa)**	**pI**
*PGRP-LB*	KC355211	scaffold1556	216	3	-	no	*D. melanogaster*	58%	24.01	6.03
*PGRP-LC*	KC355212	scaffold1031	359	3	-	no	*D. melanogaster*	59%	39.51	7.12
*GRP1*	KC355197	scaffold991	550	10	+	no	*L. migratoria*	51%	62.34	5.44
*GRP2*	KC355198	scaffold5509	499	6	+	no	*R. prolixus*	57%	56.70	6.84
*GRP3*	KC355199	scaffold991	579	14	+	no	*R. prolixus*	60%	65.83	5.69
*GRP4*	KC355200	scaffold2822	362	11	-	no	*L. migratoria*	57%	41.89	5.97
*GRP5*	KC355201	scaffold1504	366	9	-	no	*L. migratoria*	57%	42.15	5.01
*GRP6*	KC355202	scaffold991	156	3	+	no	*B.mori*	64%	17.64	5.29
*GRP7* (partial)	KC355203	scaffold412	455	16	+	no	*R. prolixus*	58%	51.44	6.42

The genomic organization of exons and introns of the genes for pattern recognition proteins is predicted based on the mRNA-genome alignments at the NCBI spideyweb (http://www.ncbi.nlm.nih.gov/spidey/spideyweb.cgi). PGRP: Peptidoglycan recognition protein; GRP: β-glucan recognition protein. Locus, size and orientation indicate the location on scaffold, predicted amino acids (aa) and the transcription orientation of the genes. UTR: Untranslated regions. Molecular weight (Mw) and isoelectric point (pI) are analyzed using Compute pI/MW tool (http://web.expasy.org/compute_pi/). *D. melanogaster*, *Drosophila melanogaster*; *L. migratoria*, *Locusta migratoria*; *R. prolixus*, *Rhodnius prolixus*; *B.mori*, *Bombyx mori.*

We investigated the *N. lugens GRP* gene expressions upon bacterial infection. Their expressions were differentially affected by gram-positive and negative bacteria species. Among these genes, *GRP5* expression was significantly up-regulated following *E. coli* K12 challenge at 6 h p.i, and returned to the level of control during 12–24 h p.i, whereas *B. subtilis* was not able to increase its expression (Figure 2). Similarly, *E. coli* K12 up-regulated *GRP4* gene expression at 6 h p.i, although it was not significant, much like the variation of *GRP5* gene expression. The fact that *E. coli* K12-induced expressions appeared at the early infection stage suggests that *GRP4* and *GRP5* genes responded quickly to gram-negative bacterial infection. Despite the β-1, 3-glucan-recognition domain not being conserved in the N-terminal end of these two genes, we could not exclude the possibility that they interact with gram-negative bacteria in the N-terminal domain-independent manner. The expression of another gene, *GRP6*, was strongly increased by both *E. coli* K12 and *B. subtilis* from 6 h p.i, before it gradually decreased to 24 h p.i. This indicated that this gene expression is responsive to both gram-negative and positive bacterial infection, and may be involved in the recognition of distinct types of bacteria in innate immune responses. *GRP1* gene expression was gradually increased upon *E. coli* K12 and *B. subtilis* injection from 6 h p.i. The other *GRP* gene expressions were not significantly induced by bacteria challenges. These results suggested that *N. lugens* GRPs probably have selective affinity with different bacteria and this leads to antibacterial responses in *N. lugens*. Tissue specificity showed that *N. lugens GRP1-7* genes have low expression levels in the gut (Figure 3A), but high levels in fat body; an important immune tissue in insects. This implies that *N. lugens* GRPs contribute to defense responses against bacteria in this tissue. Some genes, namely *GRP2*, *5* and *7* also showed high expression levels in the salivary gland and carcass including head and epidermal tissues, suggesting these GRPs may play important roles in these tissues.

### Immune signaling pathway-related molecules

In insects, Toll and Imd pathways are the major innate immune signaling pathways that sense microbes in hemolymph [32]. The Toll pathway is primarily involved in the defense against fungi and gram-positive bacteria with lysine-type peptidoglycans (Lys-type PGNs) in their cell walls, while the Imd pathway responds to gram-negative bacteria and some gram-positive bacteria with *meso*-di-aminopimelic acid-type peptidoglycan (Dap-type PGNs), namely *Bacillus*[33]. The activation of the Toll pathway takes place via the binding of an extracellular ligand, Spatzle to the transmembrane receptor Toll, which triggers an intracellular signaling cascade, including the adaptor proteins dMyD88 and Tube, while the kinase Pelle leads to the proteolytic degradation of the I-κB like inhibitor Cactus and the nuclear import of the NF-κB like transcription factors Dorsal and Dif [34,35]. In the Imd pathway, a transmembrane protein PGRP-LC, is the signal receptor that triggers an intracellular signaling transduction, including Imd, Fas-associated death domain protein (FADD), Dredd, IAP2, transforming growth factor β activated kinase (TAK1), Tab2, Ubc13, and an inhibitor of nuclease factor κB kinase subunits β and γ (IKKβ and IKKγ). This results in the activation and nuclear translocation of an NF-κB like transcription factor, Relish [25]. Toll and Imd pathways ultimately regulate the microbe-induced gene expressions including various humoral immune factors, namely antibacterial peptides.

**Figure 6:**
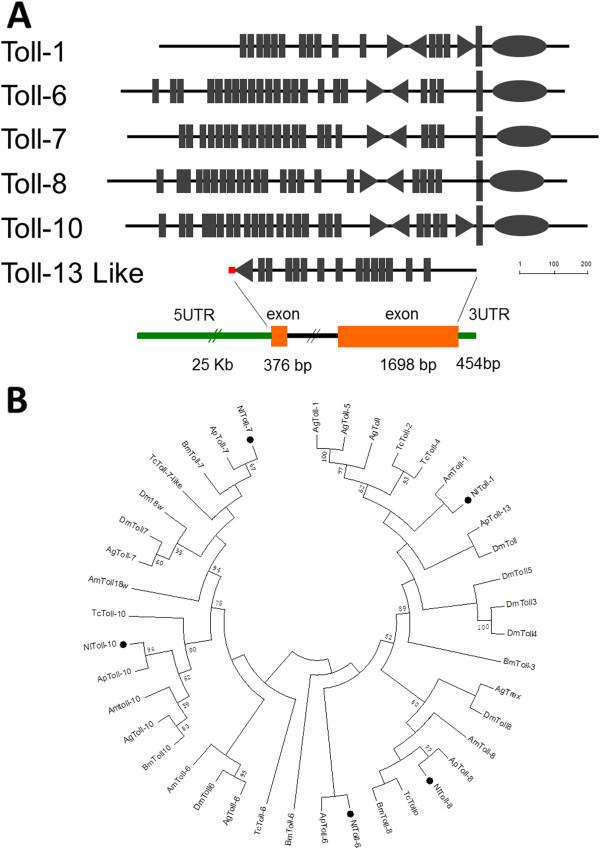
**(A) Predicted *N. lugens *Toll receptor family.** The domain organization was predicted using the SMART program (http://smart.embl.de/). The extracellular LRRs are shown as rectangles and the characteristic cysteine-rich carboxy-flanking and amino-flanking motifs are shown by triangles, while the intracytoplasmic TIR domains are shown by ellipses. The predicted structure of the *N. lugens Toll-13* like gene, including 5^′^UTR, two exons and 3′UTR, is indicated under the schematic domain representation. The size bar indicates the amino acid residues of the deduced Toll receptors. (**B**) Phylogenetic analysis of insect TIR domains. The phylogenetic tree was constructed based on the conserved TIR domains by Maximum likelihood, using the program Mega 5.05 (http://www.megasoftware.net/). The Jones-Taylor-Thornton (JTT) for amino acid substitution model was used, while a test of phylogeny was carried out using the bootstrap method with 1000 replications, bootstrap values>50% are shown on each node of the tree. Nl, *N. lugens*; Dm, *Drosophila melanogaster*; Ag, *Anopheles gambiae*; Ap, *Acyrthosiphon pisum*; Tc, *Tribolium castaneum*; Am, *Apis mellifera;* Bm, *Bombyx mori*.

The Toll receptor, as the signal transducer of the Toll pathway, plays a crucial role in insect innate immune response and embryogenesis; that is, in the establishment of dorsal-ventral polarity in the early embryo [36]. A typical Toll receptor generally contains extracellular leucine-rich repeats (LRRs) connected to a cysteine-rich domain and an intracytoplasmic Toll-interleukin homolog domain (TIR) [37]. In this study, we identified six genes coding Toll receptors in *N. lugens* genome and transcriptome datasets. These genes were designated as *N. lugens Toll-1*, *Toll-6*, *Toll-7*, *Toll-8*, *Toll-10* and *Toll-13* because of their deduced amino acids showing significant sequence similarities with their insect counterparts. The predicted proteins, with the exception of the Toll-13 like protein, consist of the extracellular LRR, transmembrane and cytoplasmic TIR domains (Figure 6A). *N. lugens Toll-13* like gene sequence was obtained from both of the predicted genomic CDS and transcriptome datasets which showed the identical coding sequence, and whose deduced protein lacked the transmembrane region and the conserved TIR domain, but had a putative signal peptide sequence. This suggests that it is a secrete-type protein. *N. lugens* genome information predicted that the *Toll-13* like gene contains two exons flanked by the 5^′^ and 3^′^ untranslated regions (UTR5 and UTR3), indicating a complete coding sequence (Figure 6A). An additional 3^′^ RACE experiment confirmed that the *Toll-13* like gene contains the full-length encoding sequence. *N. lugens Toll* genes are located in different scaffolds (Table 2). *Toll-7* and *Toll-10* are intronless, while *Toll-1*, *Toll-8*, *Toll-6*, and *Toll-13* like genes contain six, three, two, and two exons respectively.

**Table 2:** **The genomic prediction of *N. lugens *Toll family**

**Predicted gene**	**GenBank ID**	**Locus**	**Size (aa)**	**Exon**	**Orientation**	**UTR**	**LRR region**	**Transmemebrane** **and LIR**	**Best match**	**Similarity**	**Mw (KDa)**	**pI**
*Toll-1*	KC355234	scaffold1767	1156	6	-	no	14	have	*P. h. corporis*	61%	131.3	6.16
*Toll-6*	KC355235	scaffold1818	1254	2	-	no	21	have	*T. castaneum*	85%	142.5	5.86
*Toll-7*	KC355236	scaffold1910	1325	1	+	no	21	have	*P. h. corporis*	79%	150.8	6.06
*Toll-8*	KC355237	scaffold90	1296	3	+	no	21	have	*P. h. corporis*	81%	147.7	5.48
*Toll-10*	KC355238	scaffold569	1302	1	+	no	23	have	*P. h. corporis*	73%	146.4	5.57
*Toll-13*	KC355193	scaffold2123	691	2	-	have	14	no	*A. mellifera*	67%	77.13	5.22

The genomic organization of exons and introns of the genes for pattern recognition proteins is predicted based on the mRNA-genome alignments at the NCBI spideyweb (http://www.ncbi.nlm.nih.gov/spidey/spideyweb.cgi). LRR: leucine-rich repeats; TIR: Toll-interleukin homolog domain. Molecular weight (Mw) and isoelectric point (pI) are analyzed using Compute pI/MW tool (http://web.expasy.org/compute_pi/). *P. h. corporis*, *Pediculus humanus corporis*; *T. castaneum***,***Tribolium castaneum*; *A. mellifera*, *Apis mellifera.*

The TIR domain is highly conserved in insect and mammalian Toll families and has a more reliable determination of phylogeny than the extracellular LRR regions [38]. With this in mind, we constructed a phylogenetic tree with the TIR domains using the program Mega 5.05 (http://www.megasoftware.net/). The result showed that insect *Toll* receptors analyzed in this study form five major clusters, *Toll-1-5*, *Toll-6*, *Toll-7*, *Toll-8*, and *Toll-10* (Figure 6B). *N. lugens Tolls* are distributed in each cluster and are closely related to *Apis mellifera Toll-1*, *Acyrthosiphon pisum Toll-6*, *Toll-7*, *Toll-8*, and *Toll-10*, individually, suggesting that most *N. lugens Tolls* have the most closely phylogenetic relationship with those counterparts from *A. pisum*.

We investigated *Toll* gene expressions upon bacterial infection. *E. coli* K12 significantly increased the transcript levels of *Toll-1* and *Toll-13* genes, while *B. subtilis* slightly increased their transcript levels during 6–24 h p.i (Figure 2), suggesting that these two Toll receptors responded to the *E. coli* K12 challenge. Bacteria injection did not change *Toll-6*, *Toll-7*, *Toll-8*, and *Toll-10* gene expressions (data not shown).

*N. lugens Toll* genes showed distinct tissue-specific expression patterns in the 5th instar nymphs (Figure 3B). Their transcripts, with the exception of *Toll 8*, were detected at high levels in the salivary gland. *Toll 6* exhibited an exclusive expression in the salivary gland among the test tissues. *Toll 1*, *Toll 7*, *Toll 10*, and *Toll 13* genes also had the significantly high expression levels in the salivary gland, followed by the fat body and carcass. *Toll 8* gene expression is somehow different, with transcripts detected at high levels in the fat body, followed by the carcass.

### Signaling modulation-related molecules

Prophenoloxidase (proPO) activation cascade is one of the major innate immune responses in arthropods, and is similar to the blood clotting system and the complement system of vertebrates. This cascade initiates the binding of pattern recognition proteins to microbe-derived molecules, such as LPS, β-1, 3-glucan and PGN, which triggers a serine protease cascade in the hemolymph [39]. The final step in this cascade is the conversion of inactive proPO to active phenoloxidase (PO) by clip-domain serine proteases, which leads to melanization responses for the removal of invaded pathogens [40]. In arthropods, clip-domain serine proteases (CLIPs) play an important role in mediating innate immunity, namely proPO activation cascade, hemolymph clotting and embryonic development [41]. CLIPs feature at least one regulatory clip domain at the amino-terminus, and a catalytic serine protease domain at the carboxyl-terminus [42,43]. Each clip domain contains six conserved cysteine residues which form three disulfide linkages.

**Figure 7:**
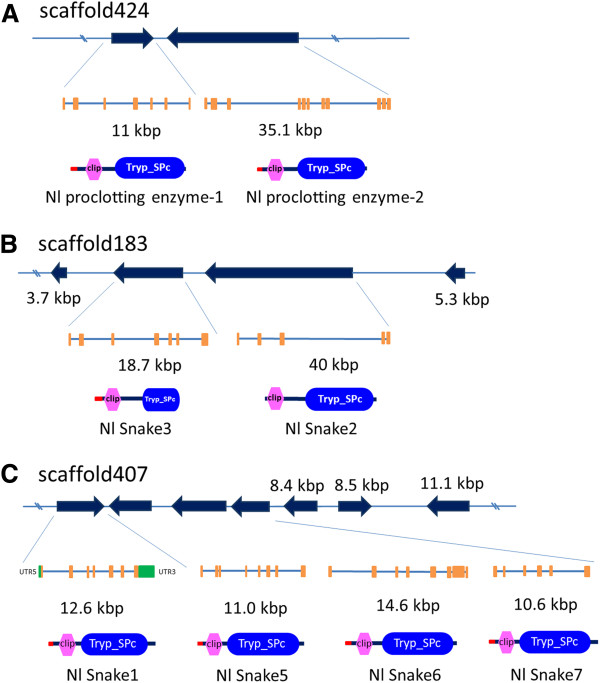
**Structure and location of *N. lugens CLIP *genes on scaffolds.** (**A**) *proclotting enzyme-1* and *proclotting enzyme-2* genes; (**B**) *snake-2* and *snake-3* genes; (**C**) *snake-1* and *snake5-7* genes. The black arrows indicate the transcription orientations and gene sizes on scaffolds. The exons are shown with orange boxes. The schematic representation of the deduced CLIP structures is shown in the panel below. Red bars, hexagons, and oblongs indicate the putative signal peptide sequence, clip domain, and serine protease domain, respectively. The small black arrows flanking the *CLIP* genes are serine proteases without clip-domains. The size bar indicates the amino acid residues of the deduced CLIPs.

**Figure 8:**
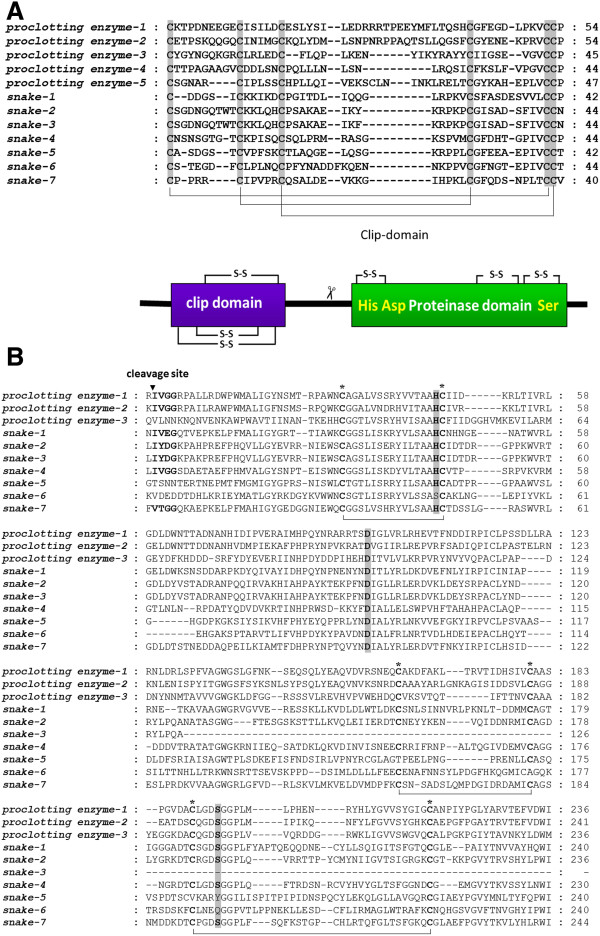
**(A) Alignments of the N-terminal clip-domains of *N. lugens *CLIPs.** (**B**) The C-terminal serine protease domains of *N. lugens* CLIPs. The CLUSTALW program was used for alignments. The gray shades indicate the conserved cysteine residues and active triad (His, Asp and Ser). The predicted disulfide linkages between conserved cysteines are shown by lines. The possible proteolytic cleavage site is indicated with an arrowhead [42,45].

Thus far, only one gene encoding CLIP (GenBank accession no. **AJ852425**) has been isolated from *N. lugens*. In this study, we identified twelve *CLIPs* by searching the *N. lugens* genomic and transcriptomic sequences. These genes distribute at seven scaffolds and their deduced amino acid sequences contain a clip domain at the N-terminus and a serine protease domain at the C-terminus (Table 3). Of these genes, five encode proclotting enzymes (*Nlproclotting enzyme1-5*) and seven encode serine protease snake-like proteins (*Nlsnake1-7*). The genome structure prediction showed that a pair of genes, *Nlproclotting enzyme 1* and *2* (GenBank accession no. **KC355213** and **KC355214**), were located at the scaffold424 and had the opposite transcription orientations, as well as containing 7 and 11 exons respectively (Figure 7A). Their deduced amino acids shared 67% and 97% sequence similarities with the known *N. lugens* CLIP (GenBank accession no. **AJ852425**). Similarly, two *CLIP* genes, *Nlsnake2* and *snake3* (GenBank accession no. **KC355220** and **KC355221**) were located at the scaffold183, and had the same transcription orientations (Figure 7B). They consisted of 5 and 7 exons, which were flanked by two *serine protease* genes without the clip-domain. In addition, four *CLIP* genes were located at the scaffold 407. *Snake1* gene (GenBank accession no. **KC355219**) includes 7 exons flanked by the 5^′^ and 3^′^ UTRs. *Snake5-7* genes (GenBank accession no. **KC355223**-**KC355225**) include 6–8 exons had the same transcription orientations. These *CLIP* genes were flanked by the additional three non-clip domain *serine protease* genes (Figure 7C). The typical clip domain was highly conserved in the deduced *N. lugens* CLIPs, which includes six cysteine residues that possibly form three putative disulfide linkages (Figure 8B). In addition, three amino acid residues (His, Asp and Ser), which are essential for the catalytic activity of serine proteases, were present in the C-terminal domain of CLIPs, except for *Nlsnake5* and *Nlsnake6*. Three disulfide linkages are probably formed among six cysteine residues in the serine protease domain (Figure 8B). CLIPs are typically synthesized as inactive zymogens and are required for activation by a specific proteolytic cleavage, which forms a regulatory light chain and a catalytic heavy chain [44]. A possible cleavage site was found in the junction region of the N- and C-terminal domains of the *N. lugens CLIPs* including *Nlproclotting enzyme 1–2*, *Nlsnake1-4* and *Nlsnake7* genes, thus implying that a proteolytic digestion occurs between the clip and serine protease domains in these *CLIPs* (Figure 8B).

**Table 3:** **The genomic prediction of *N. lugens *clip-domain serine proteases and serine protease inhibitors**

**Predicted gene**	**GenBank ID**	**Locus**	**Size (aa)**	**Exon**	**Orientation**	**UTR**	**Best match**	**Similarity**
**Clip domain serine proteases**								
*proclotting enzyme-1*	KC355213	scaffold424	397	7	+	no	*A.pisum*	56%
*proclotting enzyme-2*	KC355214	scaffold424	376	11	-	no	*A.pisum*	55%
*proclotting enzyme-3*	KC355215	scaffold1854	460	9	-	have	*A.pisum*	66%
*proclotting enzyme-4*	KC355216	scaffold32	535	8	+	no	*A.pisum*	62%
*proclotting enzyme-5*	KC355217	scaffold973	264	5	+	no	*D.plexippus*	68%
*serine protease snake-1*	KC355219	scaffold407	363	7	+	have	*A.pisum*	54%
*serine protease snake-2*	KC355220	scaffold183	partial	5	-	no	*T. castaneum*	51%
*serine protease snake-3*	KC355221	scaffold183	partial	7	-	no	*A.pisum*	47%
*serine protease snake-4*	KC355222	scaffold3538	546	7	+	no	*P. h.corporis*	58%
*serine protease snake-5*	KC355223	scaffold407	358	8	-	no	*A.pisum*	41%
*serine protease snake-6*	KC355224	scaffold407	378	8	-	no	*A.pisum*	45%
*serine protease snake-7*	KC355225	scaffold407	362	6	-	no	*A.pisum*	53%
**Serine protease inhibitors**								
*serpin-1*	KC355226	scaffold2106	partial	8	+	no	*C. suppressalis*	69%
*serpin-2*	KC355239	scaffold1141	402	5	+	have	*A.gambiae*	55%
*serpin-3*	KC355227	scaffold690	400	5	+	have	*T. castaneum*	53%
*serpin-4*	KC355228	scaffold1199	408	8	-	no	*A.pisum*	60%
*serpin-5*	KC355229	scaffold914	492	7	-	no	*B. mori*	73%
*serpin-6*	KC355230	scaffold3763	partial	11	-	no	*C. quinquefasciatus*	62%
*serpin-7*	KC355231	scaffold1822	505	4	+	no	*A.pisum*	57%
*serpin-8*	KC355232	scaffold1121	partial	4	+	no	*A.pisum*	83%
*serpin-9*	KC355233	scaffold1452	partial	5	-	no	*A.pisum*	64%

The genomic organization of exons and introns of the genes for pattern recognition proteins is predicted based on the mRNA-genome alignments at the NCBI spideyweb (http://www.ncbi.nlm.nih.gov/spidey/spideyweb.cgi). *A.pisum*, *Acyrthosiphon pisum*; *D. plexippus*, *Danaus plexippus*; *T. castaneum***,***Tribolium castaneum*; *P. h. corporis*, *Pediculus humanus corporis*; *C. suppressalis*, *Chilo suppressalis*; *A. gambiae*, *Anopheles gambiae*; *B. mori*, *Bombyx mori*; *C.quinquefasciatus*, *Culex quinquefasciatus*.

**Figure 9:**
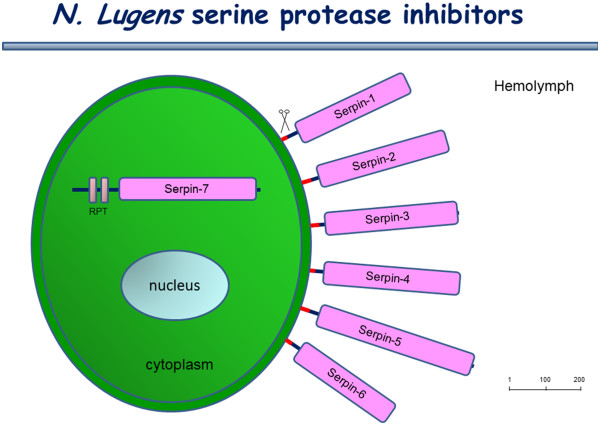
**The structure prediction and cellular distribution of the deduced *N. lugens *serpins.** Red bars and rectangles indicate the putative signal peptide and the core serpin domains, respectively. RPT indicates two N-terminal internal repeats of *serpin-7*, which may be retained in the cytoplasm. The size bar indicates the amino acid residues of the deduced serpins.

Serine protease inhibitors (serpins) present in insect hemolymph regulate the proPO activation cascade, where they function as the negative regulators to avoid excessive activation of the cascade [46]. In *Drosophila*, a well-known serpin, spn27A prevented extensive melanization by inhibiting the proPO activating protease [47]. In *Manduca sexta*, at least five serpins (serpin 1 J and 3–6) blocked the proPO activation in the cascade [48-50]. In this study, nine *serpin* genes were identified in the *N. lugens* genome. These genes distribute in different scaffolds and show high sequence similarities with insect serpins, especially the hemimetabolous species (Table 3). We designated them as *Nlserpin1-9*. A search of the *N. lugens* transcriptome determined that six genes (*Nlserpin1-6*) consisted of a predicted signal peptide sequence and a core serpin domain, suggesting that they are secreted proteins (Figure 9). Their deduced amino acids shared 53%-73% similarities with insect serpins (Table 3). The putative protein product of *Nlserpin7* gene shared a 57% similarity with *A. pisum* plasminogen activator inhibitor 1, a secreted type of serpin. Despite the significant identity, *Nlserpin7* lacked the putative signal peptide sequence. Its sequence featured two internal repeats at the N-terminus, except for a major serpin domain. The structure prediction implies that *N. lugens* serpin7 is likely to be an intracellular protein.

We analyzed the expression pattern of six *CLIP* genes in the salivary gland, fat body, gut, and carcass (Figure 3C). Their transcripts were detected at very low levels in the gut, suggesting that they probably do not function in digestion. Two genes, including *proclotting enzyme 2* and *snake 2*, exhibited the highest expression levels in the carcass among the analyzed tissues, implying that they have potential functions in the epidermis. The other *CLIPs* showed the high transcript levels in the salivary gland, suggesting that these genes might play the important roles in this tissue.

### Immune responsive effector genes

Most microbial pathogens are able to induce the expression of insect effector genes, which are generally synthesized in some specific tissues, such as fat body and hemocytes, before being released into the hemolymph where they directly attack the invaders or are involved in the proPO cascade-dependent malanization responses. The antibacterial peptides are a group of immune-responsive effectors that are regulated by the Toll and Imd signaling pathways and play important roles in the humoral defense systems of insects [51]. A variety of antibacterial peptide genes were isolated and characterized from many insect species. In this study, *defensins* are the available antibacterial peptide genes identified in the *N. lugens* genome. Several other effector genes, including *reeler*, *lysozyme*, and *NOS*, are present in the *N. lugens* genome.

**Figure 10:**
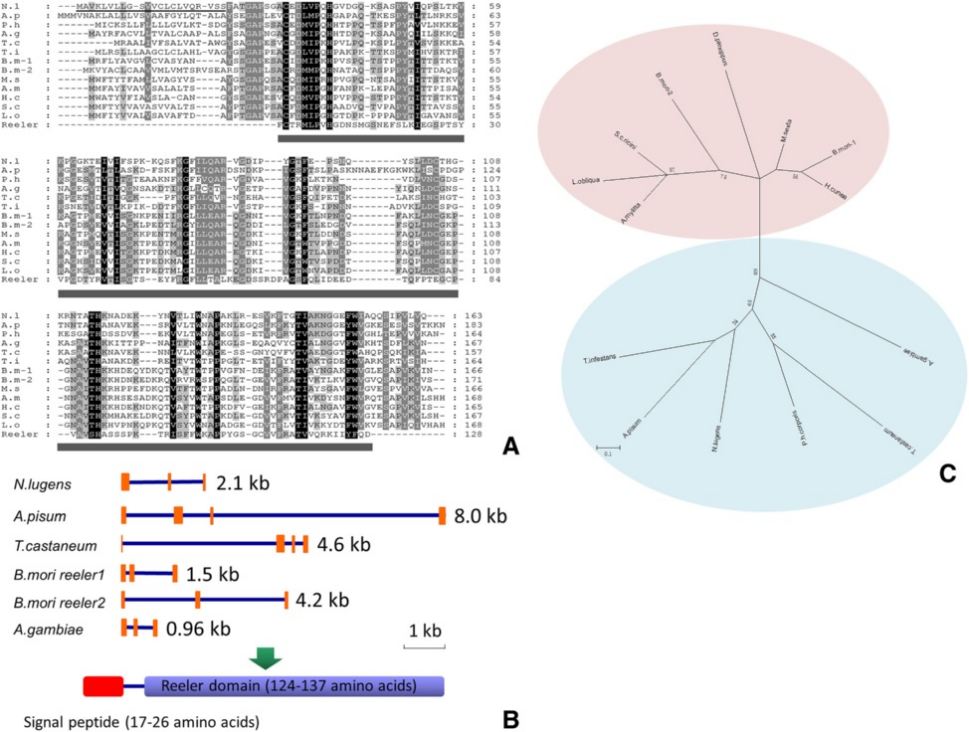
**(A) Multiple sequence alignment of Reeler proteins of several insect species.** The ClustalX program was used for alignments. The GenBank accession numbers for the sequences are as follows: *N. lugens* (NLU024648.1); *B. mori* reeler1 (HQ325059); *B. mori* reeler2 (HQ325058); *H. cunea* (AAD09280); *S. c ricini* (BAD05929); *A. mylitta* (ABG72705); *M. sexta* (AAO21507); *L. obliqua* (AAV91350), *P. h. corporis* (EEB13623); *T. infestans* (ABR27826); *A. pisum* (XP_001944294); *A. gambiae* (EAA14972); *T. castaneum* (XP_966813), and the reeler domain sequence (Pfam domain PF02014). Black and gray shading indicates the identity and high conservation of amino acids, respectively. The predicted signal peptide sequences of the deduced *N. lugens* Reeler protein is underlined. Dark gray bars under the sequences indicate the reeler domain regions. **(B)** Schematic representation of the *reeler* genes of several insect species. The orange boxes indicate the exon sizes and location of each *reeler* gene on scaffolds. The deduced Reeler proteins are shown in the below panel. Red and blue bars indicate the putative signal peptide sequence and the putative reeler domains. The size bar indicates the nucleotides of insect *reeler* genes. **(C)** Phylogenetic analysis of reeler domains of several insect species. The phylogenetic tree was constructed by Maximum likelihood using the program Mega 5.05 (http://www.megasoftware.net/). The Jones-Taylor-Thornton (JTT) for amino acid substitution model was used, a test of phylogeny was done by the bootstrap method with 1000 replications, bootstrap values>50% are shown on each node of the tree.

*Reeler* is an immune-responsive gene which mediates the nodulation response upon bacterial infection [52]. *Reeler* features a reeler domain, which was initially identified in the mouse reelin protein, a secreted glycoprotein which plays a pivotal role in the development of the central nervous system in mammals [53]. At present, *reeler* genes are well characterized only in lepidopteran insects including *Hyphantria cunea*[54], *Manduca sexta*[53], *Samia cynthia ricini*[55], *Lonomia obliqua*[56], *Antheraea mylitta*[52] and *B. mori*[57]. In this study, the *N. lugens* genome and transcriptome revealed one *reeler* gene (GenBank accession no. **KC355218**), which encodes 163 amino acid residues consisting of a putative signal peptide and a characteristic reeler domain. The predicted molecular weight of mature Reeler protein is 15.3 kDa. The reeler domain spans nearly the entire coding regions of *N. lugens reeler* (Figure 10A). The *N. lugens reeler* gene is 2.1 kb long and contains three exons. A comparison of the gene structure among several genome-available insect species revealed that the significant difference of the *reeler* gene sizes is that it varies from 0.96 kb to 8.0 kb, although these genes include no more than four exons. The deduced proteins showed that these *reelers* are composed of a signal peptide sequence with 17–26 amino acid residues and a reeler domain of 124–137 amino acid residues (Figure 10B). The phylogenetic tree shows that lepidopteran *reelers* form an independent cluster, while the *N. lugens reeler* distantly locates in another independent cluster and is closely related to the homologues of two hemimetabolous species, namely *T. infestans* and *A. pisum* (Figure 10C).

**Figure 11:**
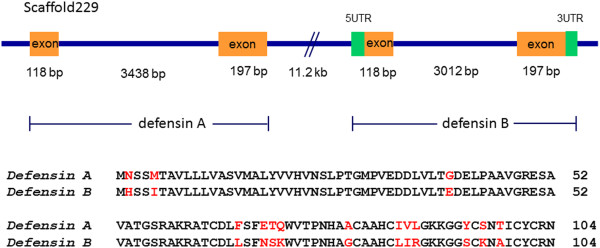
***N. lugens defensin *gene structure.** The orange boxes indicate the exon size and location of *defensin* genes on scaffold. The green boxes indicate the 5^′^ and 3^′^UTR regions. The alignment of two defensins deduced from the *N. lugens* transcriptome database is shown in the panel below. The different amino acid residues are shown in red.

We identified two *defensin* genes in the *N. lugens* genome. As an antibacterial peptide, defensin plays an important role in insect defense systems. These two *defensin* genes are located at the same scaffold. One *defensin* gene (GenBank accession no. **KC355196**) contains two exons flanked by the 5^′^ and 3^′^ UTRs; the other (GenBank accession no. **KC355195**) also contains two exons but has no 5 and 3^′^ UTR sequences (Figure 11). Accordingly, the *N. lugens* transcriptome revealed two *defensin* transcripts. Their deduced peptides include 104 amino acid residues which share 86.5% identities. The two *N. lugens defensins* showed 74% sequence similarities with *T. infestans defensin A* and *Rhodnius prolixus defensin B*, respectively. We designated them as *Nldefensin A* and *Nldefensin B* (Table 4).

**Table 4:** **The gene prediction of *N. lugens *immune responsive effectors**

**Predicted gene**	**GenBank ID**	**Locus**	**Size (aa)**	**Exon**	**Orientation**	**UTR**	**Best match**	**Similarity**	**Mw**	**pI**
*reeler*	KC355218	scaffold666	163	3	+	no	*T. infestans*	60%	15.31	9.28
*defensin B*	KC355196	scaffold229	104	2	+	have	*R.prolixus*	74%	8.35	8.31
*defensin A*	KC355195	scaffold229	104	2	+	no	*T. infestans*	74%	8.36	6.06
*c-type lysozyme*	KC355194	scaffold427	154	2	-	no	*P. h. corporis*	68%	14.68	6.64
*i-type lysozyme1*	KC355204	scaffold515	partial	3	+	no	*P. americana*	58%		
*i-type lysozyme2*	KC355205	scaffold2772	158	3	-	no	*A. pisum*	75%	15.22	5.09
*i-type lysozyme3*	KC355206	scaffold374	163	6	-	no	*P.americana*	76%	15.40	5.06
*i-type lysozyme4*	KC355207	scaffold6850	partial	3	-	no	*P.americana*	77%		
*i-type lysozyme5*	KC355208	scaffold186	166	3	-	no	*P.americana*	55%	15.89	5.29
*i-type lysozyme6*	KC355209	scaffold186	partial	3	+	no	*P.americana*	62%		
*i-type lysozyme7*	KC355210	scaffold83	176	4	+	have	*D. plexippus*	48%	17.69	7.88

The genomic organization of exons and introns of the immune responsive genes was predicted based on the mRNA-genome alignments at the NCBI spideyweb (http://www.ncbi.nlm.nih.gov/spidey/spideyweb.cgi). Molecular weight (Mw) and isoelectric point (pI) were analyzed using Compute pI/MW tool (http://web.expasy.org/compute_pi/). *T. infestans*, *Triatoma infestans*; *R. prolixus*, *Rhodnius prolixus*; *P. h. corporis*, *Pediculus humanus corporis*; *P. americana*, *Periplaneta Americana*; *A. pisum*, *Acyrthosiphon pisum*; *D.plexippus*, *Danaus plexippus*.

**Figure 12:**
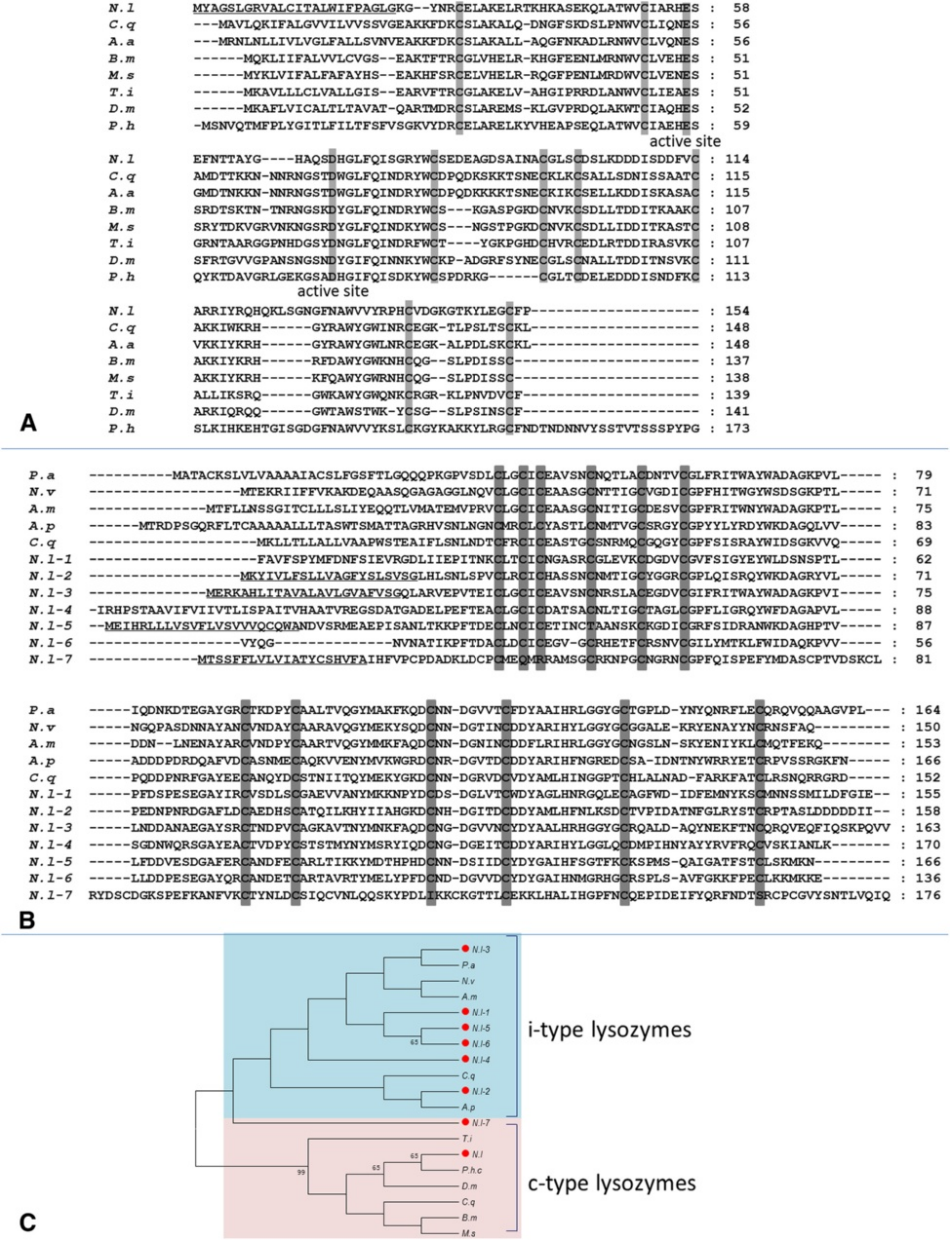
**Multiple sequence alignments of lysozymes of several insect species.** (**A**) c-type lysozyme aligments; (**B**) i-type lysozyme aligments. The ClustalX program was used for alignments. The GenBank accession numbers for the sequences are as follows: *Pediculus humanus corporis* lysozyme P precursor (EEB19248); *Bombyx mori* lysozyme precursor (AAB40947); *Manduca sexta* lysozyme (AAB31190); *Aedes aegypti* lysozyme P (EAT44944), *Triatoma infestans* lysozyme (AAP83129), *Culex quinquefasciatus* lysozyme (EDS45638), *Drosophila melanogaster* lysozyme P (AAF47452), *Periplaneta americana* i-type lysozyme (AFI81521), *C. quinquefasciatus* lysozyme i-1 (EDS32730), *Acyrthosiphon pisum* lysozyme 1-like (XP_001949318), *Nasonia vitripennis* lysozyme 3-like (XP_001600829) and *Apis mellifera* lysozyme isoform 1 (XP_393161). The predicted signal peptide sequences of lysozymes are underlined. Gray shading indicates the conserved cysteine residues and the putative catalytic sites of the enzymes. (**C**) Phylogenetic analysis of insect c- and i-type lysozymes. The phylogenetic tree was constructed by Maximum likelihood, using the program Mega 5.05 (http://www.megasoftware.net/). The Jones-Taylor-Thornton (JTT) for amino acid substitution model was used, the test of phylogeny was done by the bootstrap method with 1000 replications, bootstrap values>50% are shown on each node of the tree. *N.l, N. lugens*; *D.m*, *Drosophila melanogaster*; *A.p*, *Acyrthosiphon pisum*; *A.m*, *Apis mellifera; B.m*, *Bombyx mori*; *M. s*, *M. sexta*; *C. q*, *C. quinquefasciatus*; *T. i*, *T. infestans*; *A. a*, *A. aegypti*; *P. h. c*, *P. h. corporis*; *P. a*, *P. Americana* and *N. v*, *N. vitripennis*.

Lysozymes constitute a large and diverse family of hydrolytic enzymes. They catalyze the hydrolysis of the β-1, 4-glycosidic linkage between *N*-acetyl muramic acid and *N*-acetylglucosamine of PGN. Three major distinct lysozymes, namely the c-type (chicken type), g-type (goose type) and i-type (invertebrates), have been identified in animals [58]. The most ubiquitous of these enzymes is the c-type lysozyme, which is widely distributed in vertebrates and invertebrates. G-type lysozymes do not seem to occur in invertebrates other than some bivalve mollusk scallops [59,60] and the tunicates [61,62]. I-type lysozymes are restricted to invertebrates. All available insect genomes contain i-type lysozymes, suggesting these enzymes are widespread in insects (http://www.ncbi.nlm.nih.gov/2012.July). Despite the differences in the amino acid sequences and the biochemical properties, the functions of lysozymes were widely recognized for their contribution to antibacterial defense. In addition, some c- and i-type lysozymes function as digestive enzymes in insects, for example in *Anopheles gambiae*[63,64]. In this study, we identified one *c-type lysozyme* gene from the *N. lugens* genome and transcriptome (Table 4). The putative molecular weight of a mature *N. lugens* c-type lysozyme is 14.68 kDa. A signal peptide sequence is predicted at its N-terminus. The deduced *N. lugens* c-type lysozyme showed significant sequence similarity with the enzymes from several insect species, including dipteran, lepidopteran, hemipteran, and anoplura insects. Eight cysteine residues, which possibly form intramolecular disulfide bridges and two potential catalytic sites, namely glutamic acid and aspartic acid residues, are highly conserved in these enzymes. This may be important for the structural stability, as well as for the enzymatic activity of lysozymes (Figure 12A). Thus far, the presence of multiple i-type lysozymes has only been reported in a few mollusk species [6,65-68], as well as the mosquito *A. gambiae*[64] and the medial leech *Hirudo medicinalis*[69]. In this study, seven *i-type lysozyme* genes were identified in *N. lugens* and designated as *Nli-lysozyme1-7*. Their deduced sequences showed high similarities with the homologues from *Periplaneta americana* (Neoptera), *Nasonia vitripennis*, *Apis mellifera*, *Acyrthosiphon pisum* and *Culex quinquefasciatus* (Figure 12B). The putative signal peptides were present in the deduced amino acid sequences of *N. lugens i-type lysozyme-2*, *3*, *5*, and *7*. The protein products of *N. lugens i-type lysozyme-2*, *3* and *5* were predicted to have calculated isoelectric points (pI) of around 5.0, and molecular weights of 15–16 kDa; while *N. lugens i-type lysozyme-7* has a molecular weight 17.69 kDa heavier than the others, and is seemingly a basic enzyme with the pI of 7.88. *N. lugens i-type lysozyme-1*, *4*, and *6* did not show the signal peptide sequences, due to their incomplete sequences. Twelve cysteine residues were highly conserved in these deduced i-type lysozymes with the exception of the *N. lugens* i-type lysozyme 7*,* which contained eight cysteine residues. Reduction of disulfide bridges decreases the antibacterial activity of lysozymes [70]. The catalytic sites, glutamic acid and aspartic acid residues are not conserved in these enzymes. Whether these i-type lysozymes are inactive, or whether the glutamic acid and aspartic acid residues are necessary for their enzymatic activity, is not clear. Zavalova *et al.*[71] proposed evidence for a non-enzymatic antibacterial mode of action of lysozyme in invertebrates, as high antimicrobial activity was detected in a heat-treated lysozyme which lacked glycosidase activity towards both *Micrococcus luteus* and *E. coli*. Similarly, Cong *et al.*[72] have very recently indicated that the sea cucumber i-type lysozyme has both enzymatic and non-enzymatic antibacterial action. The precise function of *N. lugens* lysozymes remains a mystery. We compared the phylogenetic relationship of these distinct *lysozyme* genes with several insect species. *C* and *i-type lysozymes* form two independent clusters, respectively (Figure 12C). In the *c-type lysozyme* cluster, the *N. lugens* gene is closely related to the homologue of *Pediculus humanus corporis*, a hemimetabolous species. In the *i-type lysozyme* group, while *N. lugens lysozyme-1*, *5*, and *6* are clustered together and more closely related to *N. lugens lysozyme-3* than *lysozyme-2*, the *N. lugens lysozyme-7* is distantly located from the other *N. lugens lysozyme* genes.

*N. lugens defensin A* and *defensin B* gene expressions were strongly induced by both *E. coli* k12 and *B. subtilis* from 6–12 h p.i, while *reeler* gene expression was significantly up-regulated by the *E. coli* k12 challenge, but seemed not to be induced by *B. subtilis* (Figure 2)*.* We also analyzed the *N. lugens lysozyme* gene expression upon bacterial infection (Figure 2). *C-type lysozyme* gene expression was strongly induced by *E. coli* k12 from 12 h p.i and decreased at 24 h p.i, whereas its expression was notably decreased by *B. subtilis* injection at 6 h p.i, before it gradually increased from 12 h p.i and recovered to the constitutive level at 24 h p.i. The *i-type lysozyme-1* gene exhibited a different expression pattern. *E. coli* k12 and *B. subtilis* did not rapidly increase *i-type lysozyme-1* gene expression levels upon infection, but slowly up-regulated its expression levels at 24 h p.i. Several other *N. lugens i-type lysozyme* genes also appeared to cause a similar inducible expression pattern (data not shown). The results suggest that these *N. lugens* effector gene expressions are responsive to foreign pathogen infection.

*N. lugens defensin* genes showed very high expression levels in salivary glands of the 5th instar nymphs. Their transcripts were also detected at relatively high levels in the fat body followed by the gut, although extremely low levels were found in the carcass (Figure 3D). *Reeler* gene expression showed different tissue specificity; the transcripts of which were detected at much higher levels in the salivary gland and carcass than in the fat body, although the lowest levels were found in the gut suggesting this *reeler* gene may not contribute to the gut immunity. The *c-type lysozyme* gene displayed an exclusive expression in the salivary gland. *I-type lysozyme* genes showed similar expression patterns, with their transcripts exhibiting their highest levels in the salivary gland followed by the fat body, while the lowest levels were found in the gut. The fat body is thought by many to represent important immune-related tissues in insects. However, in this study, our findings indicate that the salivary gland is more likely to be the most important tissue with regards to immune defense responses in *N. lugens*.

### Development and sex-specific expression

**Figure 13:**
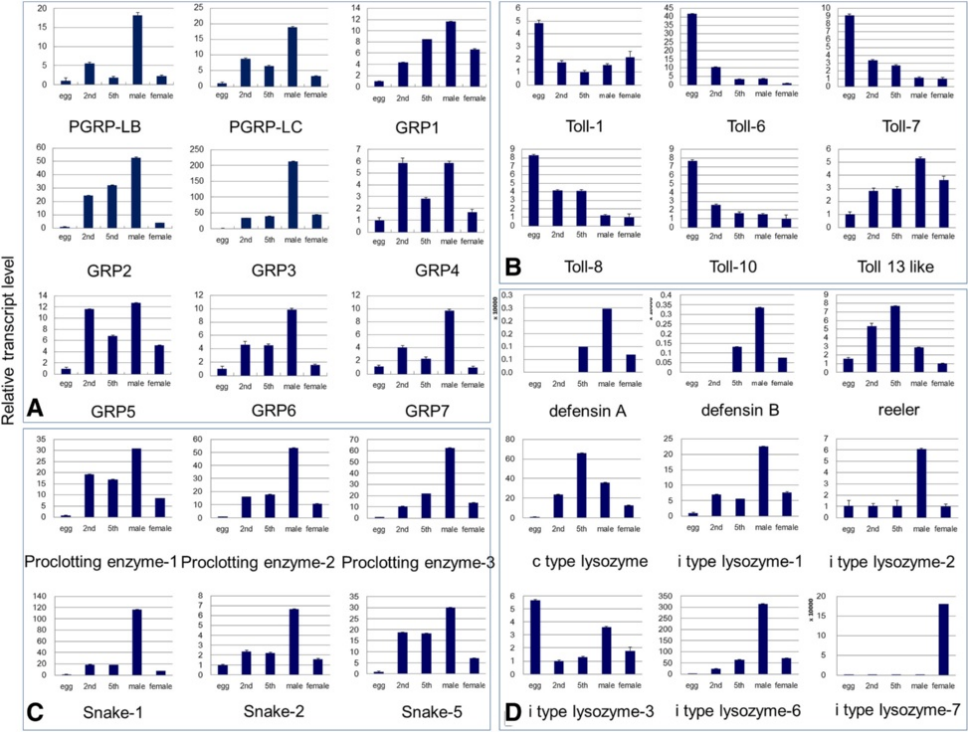
**Developmental stage- and sex-specific expression of immune-related genes in *N. lugens *Total RNA was extracted from eggs, 2nd instar nymphs, 5th instar nymphs, female adults and male adults, individually.** First-strand cDNA (20 ng) was analyzed in each qRT-PCR reaction. The reactions were performed with specific primers for amplifying (**A**) *PGRP/GRP* genes; (**B**) *Toll* genes; (**C**) *CLIP* genes; and (**D**) immune effector genes. The relative expression levels of each gene in each developmental stage or sex were normalized using the *N. lugens* 18 s rRNA threshold cycle (Ct) values that were obtained from reactions run on the same plate. In each assay, the expression level was normalized to the lowest expression level, which was arbitrarily set at one. Three technical replication (n=3) was conducted and the ΔΔCt method was used to measure the relative transcript levels in each treated sample.

In our previous study, we obtained *N. lugens* development and sex-specific expression profile data, including eggs, 2nd instar nymphs, 5th instar nymphs, female and male adults [6]. In this study, we focused on some immune-related genes and analyzed their expressions in the different developmental stages and sexes. *N. lugens PGRP* and *GRP* genes showed much higher expression levels in male adults than in female adults (Figure 13A). These genes also had relatively high expression levels in 2nd instar and/or 5th instar nymphs, although extremely low levels were found in eggs. Similarly, *N. lugens CLIP* genes also had significantly high expression levels in male adults when compared to the female adults (Figure 13C). Their transcripts were detected in nymphs, but were barely detectable in eggs. Several immune responsive effector genes exhibited different expression patterns. Two *defensin* genes possessed the identical expression pattern; while their transcripts were detected at the highest levels in male adults followed by the 5th instar nymphs, but were hardly detected in the eggs or the 2nd instar nymphs (Figure 13D). The *reeler* gene showed a distinct expression pattern, with the maximum transcript levels being detected in the 5th instar nymphs followed by the 2nd instar nymphs. However, low transcript levels were observed in eggs and adults. The *c-type lysozyme* gene showed a significantly high expression level in the 5th instar nymphs, while the *i-type lysozyme-3* gene had the highest expression level in eggs. Several other *i-type lysozyme* genes (*1*, *2*, and *6*) displayed a similar expression pattern, and their transcripts were detected at the highest levels in male adults. The *i-type lysozyme-7* gene had a completely different expression pattern, with transcripts exclusively detectable in female adults. *Toll* genes including *Toll-1*, *6*, *7*, *8* and *10* showed the highest expression levels in eggs; in contrast, the *Toll-13 like* gene had the lowest expression level in eggs (Figure 13B). The fact that the significantly high expressions appeared in eggs, that is *Toll* genes and an *i-type lysozyme* gene, suggests that they may function not only in immunity but also in embryogenesis and development. It is interesting that the majority of *N. lugens* immune-related genes had a common high expression pattern in male adults but low levels in female adults. In *N. lugens*, female adults possess many more abundant microbial symbionts than do male adults. Our findings indicate a possible immune strategy whereby female adults reduce their immune capabilities to maintain the microbial symbionts in order to meet the requirements of nutrition, development, and reproduction.

### A comparison of immune-related genes among insect species

In this study, the genome- and transcriptome-wide analysis revealed an intact innate immune network presenting in *N. lugens*. This network included the abundant pattern recognition proteins, signal transduction components involved in Toll, Imd and JAK/STAT pathways, modulation molecules in proPO activating cascade and immune responsive effectors. Comparative genome data showed that the key pattern recognition, signal transduction and modulation molecules are common in several insect species; however, the components of antibacterial peptides are different (Table 5). Antibacterial peptides play important roles in the humoral defense systems of insects. The well-known *attacin*, *cecropin*, *gloverin*, *lebocin* and *moricin* in lepidopteran insects and *diptericin*, *drosocin*, *drosomycin*, *metchnikowin* and *nuecin* in dipteran insects, are absent in the *N. lugens* genome. *Defensins* are the unique antibacterial peptide genes available in the *N. lugens* genome. A lack of most antibacterial peptides may be an effective strategy by which to maintain symbiotic systems in *N. lugens.*

**Table 5:** Immune-related genes in several insect species

**Functional classidication**	**Gene**	***N.lugens***	**A. pisum**	**D. melanogaster**	**A. gambiae**	**A. mellifera**	**B. mori**
Pattern recognition molecules	*PGRp*	2	0	13	7	4	12
	*βGRP/GNBP*	7	1	3	7	2	4
	*C-type lectin*	9	10	34	25	10	21
	*hemocytin*	1	1	1	0	1	2
	*hemolin*	0	0	0	0	0	1
	*galectin*	2	1	6	8	2	4
	*dscam*	9	1	1	1	1	1
	*Draper*	1	1	1	1	1	1
	*Eater*	1	0	1	1	0	0
	*toll*	6	5	9	10	5	14
Toll pathway	*cactus*	1	1	1	1	3	1
	*myD88*	2	0	1	1	1	1
	*spatzle*	8	4	6	6	2	3
	*pelle*	1	1	1	1	1	1
	*tube*	1	1	1	1	1	1
	*Dorsal/Dif*	1	1	2	1	2	1
	*tollip*	1	1	1	2	1	2
	*dred*	1	0	1	1	1	1
Imd pathway	*imd*	1	0	1	1	1	1
	*relish*	1	0	1	1	2	1
	*caspar*	3	2	2	1	2	1
	*IKK*	2	1	2	2	2	2
	*Tak1*	1	0	2	1	1	1
	*IAP2*	1	1	1	1	1	1
	*Ubc13*	1	1	1	1	1	1
	*TRAF*	2	2	2	1	2	1
	*Tab2*	1	1	1	1	1	1
	*Hopscoch*	1	1	1	1	1	1
JAK-STAT pathway	*PIAS*	1	12	1	1	2	1
	*SOCS*	5	5	3	1	4	3
	*STAT*	1	2	1	2	1	1
	*Domeless*	1	1	1	1	1	1
	*Clip-domain protease*	12	6	37	41	18	15
proPO cascade	*Serpin protease inhibitor*	9	14	30	17	5	26
	*Lysozyme*	8	3	17	8	3	4
Immune-responsive effector	*Reeler*	1	2	2	2	1	3
	*Defensin*	2	0	1	4	2	1
	*Attacin*	0	0	4	1	0	2
	*Cecropin*	0	0	4	4	0	13
	*Diptericin*	0	0	2	0	0	0
	*Drosocin*	0	0	1	0	0	0
	*Drosomycin*	0	0	7	0	0	0
	*Gloverin*	0	0	0	0	0	4
	*Lebocin*	0	0	0	0	0	1
	*Metchnikowin*	0	0	1	0	0	0
	*Moricin*	0	0	0	0	0	1
	*Nuecin*	0	0	0	0	0	1
	*NOS*	1	1	1	1	1	2

The number of immune-related genes in insect species was obtained from NCBI databases (http://www.ncbi.nlm.nih.gov/2012.July) coupled with the available genome databases of the insect species: *A. pisum* (http://www.inra.fr/aphidbase/); *B. mori* (ftp://silkdb.org/pub/release_2.0/); *D. melanogaster* ftp.flybase.org/genomes/Drosophila_melanogaster/dmel_r5.27_FB2010_04/); *A. gambiae* (ftp.vectorbase.org/public_data/organism_data/agambiae/Geneset/) and *A. mellifera* (hymenopteragenome.org/drupal/sites/hymenopteragenome.org.beebase/files/data/).

A genome-wide comparison of two hemimetabolous species, *N. lugens* and *A. pisum*, revealed that the major signal transducers in the Imd pathway including *IMD*, *Dredd* and *Relish* are lacking in the *A. pisum* genome [73], while the corresponding components are conserved in the *N. lugens* genome. As pattern recognition proteins, PGRPs are required to trigger the signal transduction via the Toll and Imd pathways in insects. Two *PGRP* genes were identified in the *N. lugens* genome. In contrast, the *A. pisum* genome lacked the *PGRP* sequence information. *Eater* is another pattern recognition receptor for binding a broad range of bacterial pathogens and mediating phagocytosis in *Drosophila* cellular immune responses [74]. An *eater* gene is identified in the *N. lugens* genome, but not detected in the *A. pisum* genome. In addition, the key signal transducer *myd88* in Toll pathway and antibacterial peptide genes were not found in the *A. pisum* genome. The genomic comparison between the two hemimetabolous insect species showed that *N. lugens* seemed to own a more comprehensive and complex innate immune system than *A. pisum*.

## Conclusions

A number of immune-related genes that are emerging in *N. lugens* constitute an integrated picture of the immune network, which provides the valuable clues for a better understanding of the immunological process under physiological and pathogenic conditions in this hemimetabolous insect. This immune system may primarily defend not only foreign pathogens, but is also designed to tolerate non-pathogenic microorganisms, such as microbial symbionts. In addition, the immune system may play important roles in the development, reproduction, and virus transmission of *N. lugens*. The expression specificity and biological function of additional genes identified in this study will need to be further elucidated. This would be useful for clarifying the detailed physiological and immunological mechanisms in *N. lugens* and could provide potential targets for this pest management in the future.

## Methods

### Insects

The *N. lugens* strain was originally collected from a rice field located in the Huajiachi Campus of Zhejiang University, Hangzhou, China. The insects used in this experiment were the offspring of a single female and were reared at 27±0.5°C with 70% humidity on rice seedlings (Xiushui 128) under a 16:8 h light:dark photoperiod. *N. lugens* eggs, 2nd instar nymphs, 5th instar nymphs, female and male adults were used for analyzing the development and sex-specific gene expressions.

### Immunization and collection of tissues

*N. lugens* 5th instar nymphs were anesthetized with carbon dioxide for 5–10 s at PCO_2_ = 5 mPa. The nymphs were immunized by microinjection of heat-killed *E. coli* K12 (gram-negative bacteria)or *Bacillus subtilis* (gram-positive bacteria) (5×10^7^ cells suspended in 10 ml of PBS) using the FemtoJet Microinjection System (Eppendorf, North America). Nymphs were collected at 6, 12 and 24 h after the microinjection in order to analyze the bacteria-induced gene expressions.

For tissue extraction, the 5th instar nymphs were dissected under a Leica S8AP0 stereomicroscope. The tissues including fat body, gut, salivary gland and the remaining carcass were isolated and quickly washed in a diethylpyrocarbonate (DEPC)-treated PBS solution (137 mM NaCl, 2.68 mM KCl, 8.1 mM Na_2_HPO_4_, 1.47 mM KH_2_PO_4_, pH 7.4). As the quantity of an individual nymph is extremely low, each tissue from 100 nymphs was pooled into one sample individually and was immediately frozen at −80°C.

### Identification of Immune-related genes from *N. lugens* genome and transcriptomes

The available immune-related gene sequences from other insect species were used as references to screen the *N. lugens* genomic (unpublished) and transcriptomic databases [6,7]. The candidates of *N. lugens* immune-related genes were confirmed by searching the BLASTX algorithm against the non-redundant (nr) NCBI nucleotide database using a cut-off E-value of 10^-5^. The genomic organization of exons and introns of the immune-related genes was predicted based on the mRNA-genome alignments at the NCBI spideyweb (http://www.ncbi.nlm.nih.gov/spidey/spideyweb.cgi). The deduced protein domains and signal peptides were determined by using Pfam (http://www.sanger.ac.uk/Software/Pfam/), SMART (http://smart.embl.de/) and InterProScan (http://www.ebi.ac.uk/Tools/pfa/iprscan/). Molecular weight and isoelectric point were analyzed via Compute pI/MW tool (http://web.expasy.org/compute_pi/). Immune-related genes in the genomes of the several other insect species were investigated for *Acyrthosiphon pisum* (http://www.inra.fr/aphidbase/), *Drosophila melanogaster* (ftp.flybase.org/genomes/Drosophila_melanogaster/dmel_r5.27_FB2010_04/), *Apis mellifera* (hymenopteragenome.org/drupal/sites/hymenopteragenome.org.beebase/files/data/), *Anopheles gambiae* (ftp.vectorbase.org/public_data/organism_data/aaegypti/Geneset/) and *Bombyx mori* (ftp://silkdb.org/pub/release_2.0/).

### Phylogenetic analysis

The functional domains of the deduced *N. lugens* immune-related proteins were aligned with the best-matched orthologs of other insect species using Clustal X program [75]. The phylogenic trees were constructed by Maximum likelihood using the program Mega 5.05 (http://www.megasoftware.net/). Orthologous relationships were determined using the bootstrap analysis with values of 1000 trials.

### Quantitative real-time PCR (qRT-PCR) analysis

Total RNA was isolated from *N. lugens* specimens using the SV Total RNA Isolation System (Promega). The concentration of RNA was adjusted with DEPC-treated H_2_O to 1 μg/μl, and 1 μg of RNA was reverse-transcribed in a 10 μl reaction using the ReverTra Ace® qPCR RT Master Mix with gDNA Remover Kit (ToYoBo). qRT-PCR was performed on an BIO-RAD CFX96™ Real-Time System (Bio-Rad) using the iQ™ SYBR Green® Supermix Kit (Bio-Rad), according to the manufacturers’ instructions. The first-strand cDNA (2 μl) and the no-template control (NTC, 2 μl) were used as templates for three technical replication assays in each 20 μl reaction mixture under the following conditions: denaturation at 95°C for 2 min, followed by 40 cycles of 95°C for 15 s and 60°C for 30 s. Fluorescence of PCR products was detected by adding a heat-dissociation protocol (temperature range, 65 to 95°C) during the last step of each cycle. Following amplification, melting curves were constructed and data analysis was performed on Bio-Rad CFX Manager 2.1 software. Specific primers are shown in Additional file 1: Table S1. As an internal control, the expression of *N. lugens 18 s rRNA* gene (GenBank accession no. JN662398) was analyzed using the following primers: 5^′^-CGCTACTACCGATTGAA-3^′^ (sense primer) and 5^′^-GGAAACCTTGTTACGACTT-3^′^ (antisense primer). The specificity of the primers was confirmed using NCBI BLAST algorithms (http://www.ncbi.nlm.nih.gov/). The results were standardized to the expression level of *N. lugens 18 s rRNA*. An NTC sample was run to detect any contamination and to determine the degree of dimer formation. The ∆ ∆ C_t_ method was used to analyze the relative differences in the transcript levels.

## Abbreviations

PGN: Peptidoglycan; PGRP: Peptidoglycan recognition protein; βGRP: β-glucan recognition protein; GNBP: Gram-negative binding protein; CLIP: Clip-domain serine proteases; bp: Base pair; CDS: Coding sequence; RACE: Rapid amplification of cDNA ends; UTR: Untranslated region; PO: Phenoloxidase; proPO: Prophenoloxidase; YLS: Yeast-like symbiont; Imd: Immunodeficiency; JAK-STAT: Janus kinase/signal transducers and activators of transcription; DEPC: Diethylpyrocarbonate; NTC: No-template control; LRR: Leucine-rich repeats; TIR: Toll-interleukin homolog domain; qRT-PCR: Quantitative real-time PCR; Ig: Immunoglobulin; Dscam: Down syndrome cell adhesion molecule

## Competing interests

The authors declare that they have no competing interests.

## Authors’ contribution

CXZ and YYB conceived and designed the experiments. YYB analyzed the *N. lugens* genomic and transcriptomic data, and performed the experiments of bacteria-challenge, tissue and development-specific expressions. LYQ conducted the cDNA cloning and sequencing. DZ and LBC performed the experiments of immune effector and *CLIP* gene expressions. Jin performed the experiments of *Toll* gene expressions. LMX performed the experiments of *PGRP* and *GRP* gene expressions. JAC provided us the valuable suggestions about this work. All authors discussed the results and commented on the manuscript. All authors read and approved the final manuscript.

## Supplementary Material

Additional file 1: Table S1. Primers used in real-time qPCR for immune-related gene specific expressions. (DOCX 22 kb) Click here for file
