# Supplementary material for: The genome- and transcriptome-wide analysis of innate immunity in the brown planthopper, Nilaparvata lugens
Source: BMC Genomics. 2013 Mar 9;14:160. doi: 10.1186/1471-2164-14-160 (PMC3616906; doi:10.1186/1471-2164-14-160)
Supplement: Additional file 1: Table S1. — Primers used in real-time qPCR for immune-related gene specific expressions. (DOCX 22 kb) [file 1471-2164-14-160-S1.docx]

Table S1. Primers used in real-time qPCR for immune-related gene specific expressions

| **Gene name** | **Forward primer** | **Reverse primer** |
| --- | --- | --- |
| *defensin A* | 5’ TTCTTCAGATCCAGTGCGGTCAC3’ | 5’ GGTCGCTACAGCCGATTCACG3’ |
| *defensin B* | 5’ TACTGCTGTTCTTCTGCTGGTT3’ | 5’ AGCTGCCTCCCTTCTTGC 3’ |
| *reeler* | 5’GGCATTCTTATGAGTTGCGGTGTT3’ | 5’CCTGTGCTTAGTGCAGAGGGTGA3’ |
| *c type lyzysome* | 5’ TATCGACAACATCAGAAGC 3’ | 5’ TCATACACTATCACCCCAA 3’ |
| *i type lyzysome-1* | 5’ GAGATGTGTGCGGTGTTTTC 3’ | 5’ AGCTGTCCCCTGTTGTGTAA 3’ |
| *i type lyzysome-2* | 5’ TGAAGATAATCCCAACAG 3’ | 5’ AGAGTACCTCAAACCAAA 3’ |
| *i type lyzysome-3* | 5’ AGGAGATGTCTGCGGCATC 3’ | 5’ TTGTACTGAGCGTCGAGCG 3’ |
| *i type lyzysome-6* | 5’ AGTCTGAGGGAGCATACCA 3’ | 5’ TTCTTCAAACACTCGGGAA 3’ |
| *i type lyzysome-7* | 5’ TTTCCTGGTTTTAGTTATCGCTA3’ | 5’ GATTGCTGAAGATTCACACATTG 3’ |
| *proclotting enzyme-1* | 5’ GACATCTGACATTGGGCTT3’ | 5’ CGTATAGTTGCGATTGCTC3’ |
| *proclotting enzyme-2* | 5’ TACGAAAATGAAAAACCGA3’ | 5’ ATTGAGAATAGCAGGACGC3’ |
| *proclotting enzyme-3* | 5’ GGAGCGCATCATCAACCAC3’ | 5’ CAACTTTCCCCAACCGACT3’ |
| *snake1* | 5’ ACTATTGGAGGGGGGGCTGACAC3’ | 5’ CCACTGATGGTAGGCGACGACGT3’ |
| *snake2* | 5’ TCGTGTAGCGGAGATAATGG3’ | 5’ GTTTGAACGGTGGCGGAGTC3’ |
| *snake5* | 5’ CGGTTTGAAGGCTTTGTTA3’ | 5’ GCTGAGCATCATCCTCGTT3’ |
| *PGRP-LB* | 5’GTGATATGTGGCGATGCCAATAT3’ | 5’GCTGTTTTGCTAGGTCCAACATC3’ |
| *PGRP-LC* | 5’ TGGTGCGAAACATCCAGGACTTC3’ | 5’ TGCGGTAGCTGTAAATGTACCTATGAG3’ |
| *GRP1* | 5’ GAATGTCAAACTGTCCCGTCAAC3’ | 5’ GAGGGTTGGTGGGGAAGTAGAT3’ |
| *GRP2* | 5’ AAGATGGCTTAGGGTATGAACGA3’ | 5’ GGTGGTAATAGGACGAGGAGGTG3’ |
| *GRP3* | 5’ GAAGCCAGCGTTGTTAGATGAGT3’ | 5’ GTTGAAGCCACAGCTCGGGAATA 3’ |
| *GRP4* | 5’TAGTGAAATCGCACGGTAACAAC3’ | 5’TGGTAGTCATACGAAGATGGTGC3’ |
| *GRP5* | 5’ CGAAACTCCTACAAAGGCAATG3’ | 5’CCTAAGGCTCTCGTCCAACTCTA3’ |
| *GRP6* | 5’ CTACGAAGTGCCATCAGCAATAA3’ | 5’ CGACTCCATCCTTCACAACATAA3’ |
| *GRP7* | 5’ GAGGAGACTTGGGTGGACATTAC3’ | 5’ TTGTGACAGTTGGCGTCGTTC3’ |
| *Toll-1* | 5’ AAACCCAAACTGGAAGTAATCG3’ | 5’ ATGTCGAACCGTGAGATGTTGT3’ |
| *Toll-6* | 5’ TACAGTCGGAGTGGTGTCGGTTTG3’ | 5’ GGATGGTCGTGTTCGTCTTCAGGT3’ |
| *Toll-7* | 5’ TGCATTATAGGGACCTGCCACA3’ | 5’ CACCAGCACCAGCTTGAACACT3’ |
| *Toll-8* | 5’ CAAACTGGTGACCCTACCTCCC3’ | 5’ GTTGACCCACTCGTCATTGAGC3’ |
| *Toll-10* | 5’ TGGCTACAACGAATCAACCAGAT3’ | 5’ TTTCACAGTCACATGCGTCAAAC3’ |
| *Toll 13-like* | 5’ ACTTCTTTCTCCGCTCACCTCCTT3’ | 5’ TTACCAGCCAACCTCAACTCTTCC3’ |
